# Severe Dengue and Dengue–Malaria Coinfection: A Case Series from a Referral Hospital in Montería, Colombia

**DOI:** 10.3390/clinpract16080150

**Published:** 2026-08-15

**Authors:** Paula A. Avilés-Vergara, Dina Ricardo-Caldera, Osnamir Elias Bru-Cordero, Juan Alberto Miranda, Angie Paola Martínez Villera, Kevin Alexander Angulo Álvarez, María Carolina Geney Caro

**Affiliations:** 1 Grupo de Investigación en Enfermedades Tropicales y Resistencia Bacteriana, Facultad de Ciencias de la Salud, Universidad del Sinú Elías Bechara Zainúm, Montería 230001, Colombia; dinaricardoc@unisinu.edu.co; 2 Dirección Académica, Universidad Nacional de Colombia, Sede de La Paz, Kilómetro 9 Vía Valledupar-La Paz, Valledupar 202017, Colombia; oebruc@unal.edu.co; 3 Programa de Medicina, Facultad de Ciencias de la Salud, Universidad del Sinú Elías Bechara Zainúm, Montería 230001, Colombia; juanmiranda@unisinu.edu.co (J.A.M.); angiemartinezv@unisinu.edu.co (A.P.M.V.); kevinangulo@unisinu.edu.co (K.A.A.Á.); mariageney@unisinu.edu.co (M.C.G.C.)

**Keywords:** severe dengue, dengue–malaria coinfection, acute febrile syndrome, mortality, Colombia

## Abstract

**Background/Objectives:** Severe dengue and dengue–malaria coinfection represent major diagnostic and therapeutic challenges in tropical endemic settings, where overlapping clinical manifestations may delay recognition of deterioration. This study aimed to describe the clinical and epidemiological characteristics of patients with severe dengue, dengue with warning signs, and dengue–malaria coinfection treated at a referral hospital in Montería, Córdoba, Colombia. **Methods:** A retrospective case series was conducted by reviewing medical records of patients diagnosed with dengue between 2018 and 2023. Cases classified as dengue without warning signs were excluded. The final analysis included patients with dengue-warning signs, severe dengue, and dengue–malaria coinfection. Dengue classification followed the 2009 World Health Organization criteria, and malaria was confirmed by thick blood smear. Sociodemographic, clinical, laboratory, geographic, and outcome-related variables were collected. Descriptive analyses were performed, and selected categorical variables were compared using Fisher’s exact test. **Results:** Fifty-three patients were included: 25 (47.17%) with severe dengue, 14 (26.42%) with dengue with warning signs, and 14 (26.42%) with dengue–malaria coinfection. Severe dengue was more frequent among females, whereas dengue with warning signs and coinfection predominated in males. Children accounted for the highest proportion of severe dengue cases, while coinfected cases were mainly distributed between childhood and adolescence. Fever was documented in all patients. Edema, elevated hematocrit, severe plasma leakage, and hemodynamic compromise were more frequent among severe dengue cases, whereas myalgia differed significantly across clinical groups. Ten deaths were recorded, six occurring in coinfected patients. **Conclusions:** Severe dengue and dengue–malaria coinfection are clinically complex conditions with overlapping manifestations and potentially fatal outcomes, highlighting the need for early recognition, differential diagnosis, and close monitoring.

## 1. Introduction

Vector-borne diseases (VBDs) remain a major global public health concern, causing substantial morbidity and mortality, particularly in tropical and subtropical regions. Among them, dengue and malaria are especially relevant because of their persistent transmission, recurrent outbreaks, and impact on vulnerable populations. Their distribution is shaped by demographic, social, and environmental conditions, while climate variability has further modified vector dynamics and extended transmission periods, increasing the risk of disease persistence and expansion into new areas [1].

In Colombia, dengue and malaria continue to pose an important public health challenge. Although they differ in ecological and biological features, processes such as unplanned urbanization, climate variability, human mobility, and population displacement have contributed to overlapping transmission scenarios in some endemic territories [2,3,4,5]. This overlap is particularly relevant in regions where both diseases coexist and where access to timely diagnosis and clinical monitoring may be limited.

Although different mosquito vectors transmit dengue and malaria, both diseases may coexist in tropical and subtropical regions where environmental conditions, human mobility, and overlapping transmission areas allow sequential or simultaneous exposure. Dengue–malaria coinfection has been reported in several endemic regions, although its frequency varies according to geographic setting, diagnostic methods, study population, and local transmission intensity [6,7]. Recent systematic reviews and regional studies suggest that coinfection is less frequent than monoinfection but clinically relevant and potentially underrecognized, particularly in settings where acute febrile illness is evaluated using limited diagnostic algorithms; reported frequencies range from approximately 2% to 7% among febrile patients in co-endemic areas [6,8,9,10].

The clinical distinction between dengue and malaria can be difficult, particularly during the early stages of illness, because both infections may present as acute febrile syndromes with nonspecific manifestations. In more severe presentations, the diagnostic challenge becomes even greater, as dengue with warning signs, severe dengue, and complicated malaria may share manifestations such as hemodynamic instability, bleeding, organ dysfunction, and other systemic complications. This overlap may delay etiological recognition and complicate therapeutic decision-making, especially in referral centers serving populations from endemic urban and rural settings [11,12].

Laboratory abnormalities commonly associated with dengue, including thrombocytopenia, alterations in leukocyte counts, and hepatic involvement, may also occur in malaria, limiting their usefulness for early etiological differentiation when interpreted in isolation [10,13]. Previous studies have shown that clinical findings alone are often insufficient to reliably distinguish between these infections, particularly in endemic settings where both diseases may coexist [6,10]. Moreover, coinfection may further complicate the clinical course by combining inflammatory, hematological, and endothelial disturbances that can amplify disease severity. This diagnostic challenge is especially relevant because delayed recognition of malaria may result in severe complications and death despite the availability of effective treatment. Therefore, in patients presenting with acute febrile illness in endemic regions, particularly those originating from malaria-endemic municipalities, clinicians should maintain a high index of suspicion for malaria even when dengue is strongly suspected [13].

In the Córdoba department, dengue is an important endemic arboviral disease, particularly in urban and peri-urban settings such as Montería. In contrast, malaria transmission is not uniformly distributed across the department but is concentrated mainly in municipalities in the southern subregions, including Tierralta, Valencia, Puerto Libertador, and Montelíbano, which have historically contributed a substantial proportion of malaria cases in Córdoba [14]. This heterogeneous territorial distribution creates clinically complex referral scenarios, as the referral hospital in Montería receives patients with dengue with warning signs and severe dengue, as well as complicated malaria cases referred from malaria-endemic municipalities. In this context, dengue–malaria coinfection may increase diagnostic uncertainty and delay appropriate management, underscoring the need for local hospital-based evidence describing the clinical behavior, severity findings, and outcomes of these patients [13].

Generating evidence from referral hospitals is particularly relevant in endemic regions, as these institutions concentrate patients with more severe presentations and provide a real-world view of the clinical burden faced by health services. In this context, describing the characteristics of patients with dengue with warning signs, severe dengue, and dengue–malaria coinfection may help improve early recognition of severity findings, support differential diagnosis in routine care, and inform clinical and epidemiological surveillance strategies in settings where both infections coexist. Therefore, this study aimed to describe the clinical and epidemiological characteristics, complications, and outcomes of patients with dengue with warning signs, severe dengue, and dengue–malaria coinfection treated at a referral hospital in Montería, Córdoba, Colombia.

## 2. Materials and Methods

### 2.1. Study Design and Setting

A retrospective hospital-based case series was conducted using a curated dengue surveillance database from the Institutional Epidemiology Office, followed by a review of the corresponding medical records. The study included patients classified as having dengue with warning signs, severe dengue, or dengue–malaria coinfection who were treated between 2018 and 2023 at Hospital San Jerónimo de Montería, Colombia.

Hospital San Jerónimo de Montería is a public State Social Enterprise (ESE) classified as a medium- and high-complexity healthcare institution. The hospital provides comprehensive health services to populations mainly from the departments of Córdoba, Sucre, and Antioquia, including individuals affiliated with the subsidized health insurance system, users from different Health Promotion Entities (EPS), uninsured low-income populations, and victims of armed conflict. This institutional profile positions the hospital as a regional referral center for the management of clinically complex infectious diseases.

### 2.2. Case Definition and Selection of Cases

The study population was identified from a dengue surveillance database provided directly by the study hospital’s Institutional Epidemiology Office. The institution had previously curated this database in accordance with its routine epidemiological surveillance procedures. It included patients classified as having dengue with warning signs, severe dengue, or dengue–malaria coinfection during the study period. Based on this database, the corresponding medical records were reviewed to extract clinical, laboratory, epidemiological, and outcome-related information. Because the objective of this study was to describe clinically complex presentations requiring hospital-based assessment and monitoring, the analysis was restricted to patients classified as dengue with warning signs, severe dengue, or dengue–malaria coinfection. Cases recorded as dengue without warning signs were not included in the database provided for this study.

Dengue diagnosis and clinical classification were established according to the 2009 World Health Organization (WHO) criteria [15]. Laboratory confirmation was based on rapid serological testing for dengue antibodies, as documented in the medical records and the institutional surveillance database. The database recorded dengue status as positive but did not consistently provide individual IgM/IgG patterns for each patient. NS1 antigen testing and molecular confirmation by reverse transcription polymerase chain reaction (RT-PCR) were not part of routine diagnostic procedures at the study hospital during the study period and were therefore not systematically performed in this cohort. Because this was a retrospective study based on routine clinical practice and secondary data, case classification relied on the diagnostic information available in the institutional medical records and surveillance database.

Dengue–malaria coinfection was defined as the simultaneous documentation of dengue and malaria during the same hospitalization for acute febrile illness, as recorded in the institutional medical records and surveillance database. Malaria diagnosis was established using the routine parasitological procedures of the institutional clinical laboratory. Malaria infection was confirmed by microscopy of a thick blood smear, and *Plasmodium* species identification was based on the corresponding peripheral blood smear report documented in the medical records. Parasite density, expressed as parasites/µL, was recorded when available.

### 2.3. Variables Collected

Sociodemographic variables, including age, sex, place of origin, and occupation, were collected from the medical records. Clinical manifestations documented during hospital care were also extracted. In addition, clinical complications, coinfections, laboratory findings available in the records, and clinical outcomes were documented.

### 2.4. Statistical Analysis

A descriptive analysis was conducted. Categorical variables were summarized with absolute frequencies and percentages, whereas quantitative variables were described using measures of central tendency and dispersion. Given the retrospective, descriptive nature of this case series and the limited sample size, statistical comparisons were considered exploratory. Comparisons between clinical groups for selected categorical variables were performed using Fisher’s exact test based on the observed frequencies. Variables directly related to the definition of severe dengue were described separately and excluded from comparative analyses. *p*-values were interpreted cautiously and used only to explore possible differences between clinical groups, not to establish inferential or causal associations.

## 3. Results

### 3.1. General Characteristics and Clinical Classification

A total of 53 patients were included in the analysis during the study period. Of these, 25 (47.17%) were classified as severe dengue, 14 (26.42%) as dengue with warning signs, and 14 (26.42%) as dengue–malaria coinfection. Among coinfected patients, *Plasmodium falciparum* was identified in 8 cases (57.1%), whereas *Plasmodium vivax* was identified in 6 cases (42.9%).

Male patients predominated in the coinfection and dengue with warning signs groups, accounting for 85.7% and 92.9% of cases, respectively. In contrast, severe dengue was more frequent among female patients, who represented 76.0% of cases in this group. The median age varied across groups. Coinfected patients had a median age of 14 years (IQR: 6–19), patients with dengue with warning signs had a median age of 18 years (IQR: 12–21), and patients with severe dengue had a median age of 12 years (IQR: 8–22).

According to age group distribution, children accounted for the highest proportion of severe dengue cases, whereas adults represented the highest proportion of dengue with warning signs cases. Coinfected cases were mainly distributed between childhood and adolescence, with 35.7% in each category. The median time from symptom onset to medical consultation was 3.5 days (IQR: 3–4) in coinfected patients, 4 days (IQR: 3–4.75) in patients with dengue with warning signs, and 4 days (IQR: 3–5) in those with severe dengue, showing a similar consultation pattern across the three groups (Table 1).

### 3.2. Geographic Distribution of Cases

The geographic distribution of cases by subregion of origin varied across the study groups. In Córdoba, San Jorge accounted for the highest number of cases (*n* = 13), followed by Alto Sinú and Sinú Medio, with 9 cases each. In San Jorge, coinfected patients represented the highest proportion of cases (7/13; 53.8%), whereas in Alto Sinú and Sinú Medio, severe dengue accounted for the highest proportion, with 6 of 9 (66.7%) and 5 of 9 (55.6%) cases, respectively.

Other subregions of Córdoba showed lower case counts. Costanera reported three cases, two of which were dengue with warning signs and one a coinfection. Bajo Sinú and Sabana each reported one case, both classified as dengue–malaria coinfection.

In Antioquia, Urabá accounted for 10 cases, most of which were classified as severe dengue (6/10; 60.0%), followed by dengue with warning signs (4/10; 40.0%). Bajo Cauca reported five cases, with an even distribution between severe dengue and dengue with warning signs (2 cases each), and one coinfected case. Valle de Aburrá reported two cases, distributed equally between severe dengue and coinfection.

**Figure 1:**
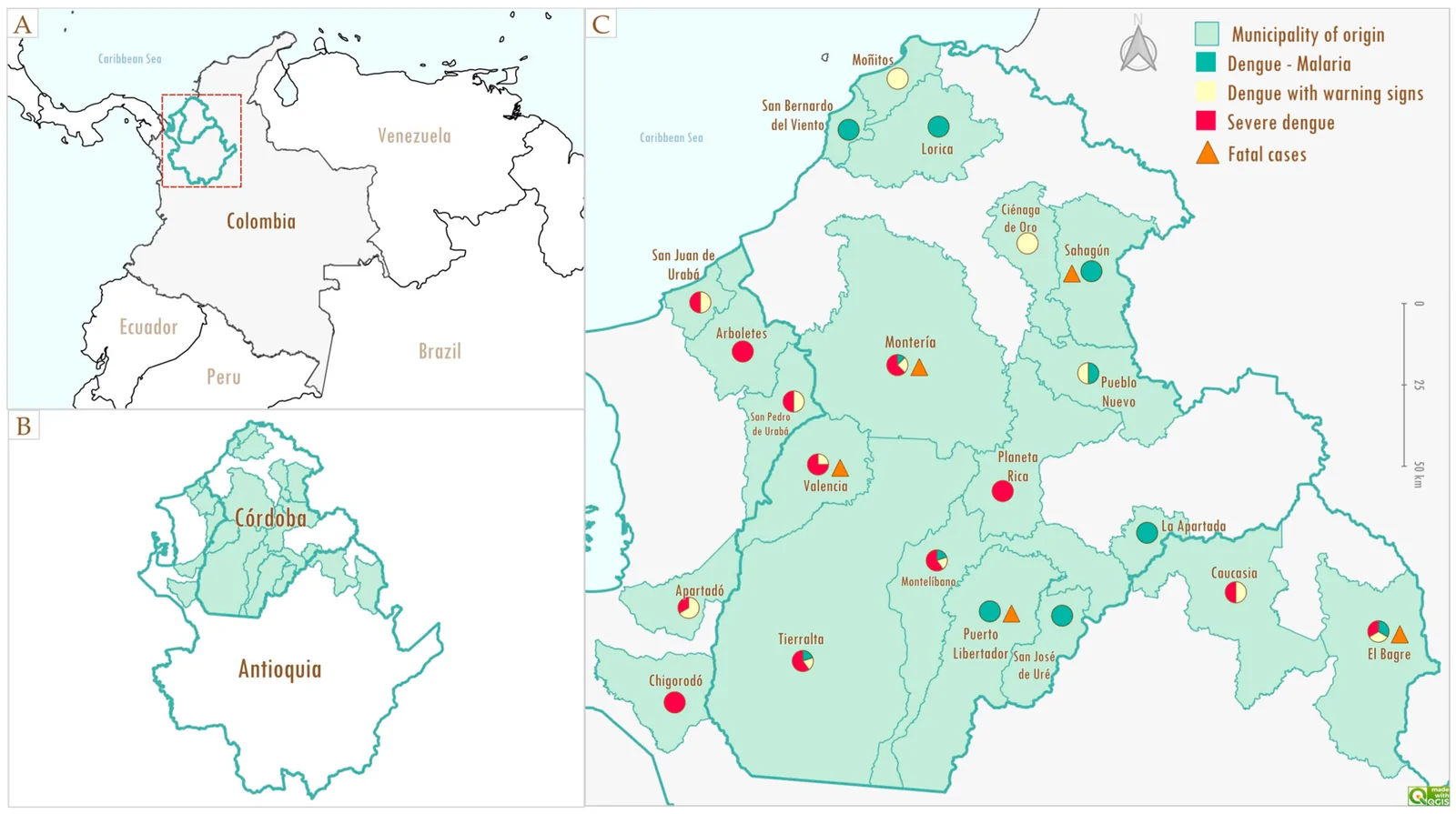
Distribution of clinically classified dengue cases and dengue–malaria coinfections in Córdoba and Antioquia. (**A**) Location of the study area in Colombia. (**B**) Departments analyzed in the study (Córdoba and Antioquia). (**C**) Municipal-level distribution of confirmed cases by clinical classification. Circles denote dengue with warning signs, severe dengue, or dengue–malaria coinfection, while triangles indicate fatal outcomes.

Figure 1 illustrates the spatial distribution of cases by municipality of origin in Córdoba and Antioquia. Cases were unevenly distributed across the study area, with a higher concentration of coinfected patients in municipalities from the San Jorge subregion and a greater frequency of severe dengue cases in Alto Sinú, Sinú Medio, and Urabá. Fatal cases were observed in both departments, with the majority in Córdoba. Overall, the observed subregional heterogeneity was characterized not only by differences in total case counts but also by distinct clinical patterns, with coinfection concentrated mainly in San Jorge and severe dengue more frequent in Alto Sinú, Sinú Medio, and Urabá (Figure 1, Table 1).

### 3.3. Clinical Manifestations and Complications

Fever was documented in all patients across the three clinical groups. Among general and systemic manifestations, loss of appetite was frequent in all groups, being reported in 85.7% of patients with dengue with warning signs, 68.0% of those with severe dengue, and 78.6% of coinfected patients. Fatigue was more common among coinfected cases (71.4%) than in dengue with warning signs (50.0%) or severe dengue (40.0%), although this difference was not statistically significant. Hypertension was infrequently documented across all groups. Because information on prior hypertension or other cardiovascular comorbidities was not consistently available in the medical records, this finding should be interpreted with caution. It may reflect pre-existing conditions rather than manifestations directly attributable to dengue or malaria.

Among neurological manifestations, somnolence and irritability were highly frequent across all clinical categories, occurring in 92.9% of patients with dengue with warning signs, 84.0% of patients with severe dengue, and 92.9% of coinfected patients. Headache and retro-orbital pain were also common, without statistically significant differences between groups. Dizziness was observed more often in severe dengue (60.0%), although no significant association was identified.

Regarding musculoskeletal manifestations, myalgia showed significant differences between groups (*p* = 0.0013), being more frequent in dengue with warning signs (92.9%) and less common in severe dengue (36.0%). Arthralgia was reported in all groups, with the highest proportion in coinfected patients (78.6%), although this difference was not statistically significant.

Among dermatological findings, rash was more frequent in severe dengue (64.0%) than in dengue with warning signs (28.6%) or in coinfected patients (35.7%), showing a borderline trend that did not reach statistical significance (*p* = 0.0747). Exanthema was observed most frequently in coinfected cases (50.0%), but differences across groups were not significant.

Gastrointestinal manifestations were common in the three groups. Abdominal pain was one of the most frequent symptoms, particularly in dengue with warning signs (92.9%), followed by coinfected patients (78.6%) and severe dengue cases (72.0%). Vomiting was also frequent across all groups, ranging from 71.4% to 78.6%. Nausea was more common among coinfected patients (71.4%), while diarrhea was more frequent in severe dengue (52.0%). Hepatomegaly was observed in more than half of the cases in all groups, occurring in 57.1% of dengue with warning signs cases, 72.0% of severe dengue cases, and 57.1% of coinfected patients. Gastrointestinal bleeding was documented in seven patients (13.2%), occurring in 24.0% of patients with severe dengue and 7.1% of patients with dengue–malaria coinfection, while no cases were observed among patients with dengue with warning signs. The available medical records did not consistently specify the type of gastrointestinal bleeding, such as hematemesis or melena. In addition, hepatic failure was documented in one fatal coinfected case. None of these gastrointestinal manifestations showed statistically significant differences.

Among hemorrhagic and hematologic findings, mucosal bleeding was frequent in all groups and was most common in severe dengue (84.0%). Elevated hematocrit was also more frequent in severe dengue (80.0%) and coinfected patients (71.4%) than in dengue with warning signs (42.9%), showing a borderline trend (*p* = 0.0778). Thrombocytopenia (<50,000/µL) was documented in nearly all patients, occurring in 100% of dengue with warning signs cases, 100% of severe dengue cases, and 92.9% of coinfected patients.

Regarding capillary leakage and hemodynamic compromise, edema showed significant differences between groups (*p* = 0.0003), with the highest frequency in severe dengue (76.0%), followed by coinfected cases (35.7%), and the lowest in dengue with warning signs (7.1%). Variables directly related to severity definition, including severe plasma leakage, hemodynamic compromise, dengue shock, and severe organ involvement, were described separately and not included in inferential comparisons. These findings were concentrated mainly in severe dengue and, to a lesser extent, in dengue–malaria coinfection (Table 2).

### 3.4. Mortality and Temporal Distribution of Cases

A total of 10 deaths were recorded during the study period, with an equal distribution between females and males (five cases each). Ages ranged from 4 to 82 years. Six fatal cases occurred in patients with dengue–malaria coinfection, whereas four corresponded to severe dengue (Table 3).

Most fatal cases originated in the Córdoba department (80%), particularly in Montería, Puerto Libertador, Sahagún, Montelíbano, and Valencia. The remaining two fatal cases were from Antioquia, corresponding to Medellín and El Bagre.

Among the six fatal coinfected cases, five were associated with *Plasmodium falciparum* and one with *Plasmodium vivax*. Common clinical manifestations documented in all fatal cases included fever, retro-orbital pain, fatigue, and severe plasma leakage. Additional case-specific findings included mucosal bleeding, thrombocytopenia (<50,000/µL), edema, hepatomegaly, hemodynamic compromise, gastrointestinal bleeding, and hepatic failure. The documented causes of death included hypovolemic shock, organ failure, hemorrhagic complications, pulmonary embolism, septic shock, and respiratory failure.

During the 2018–2023 period, dengue with warning signs exhibited a fluctuating distribution, with cases recorded in 2018, 2019, 2022, and 2023, and no cases reported in 2020 or 2021. Severe dengue also varied over time, with a marked increase in 2019, a decrease in 2020, and a subsequent rise through 2023. In contrast, dengue–malaria coinfection showed a decreasing distribution over the study period, declining from 2018 to 2023.

## 4. Discussion

The findings of this case series provide clinically relevant evidence on the epidemiological and clinical characteristics of severe dengue and dengue–malaria coinfection in patients treated at a referral hospital in Montería, Córdoba. In this study, severe dengue was more frequent among females, whereas dengue–malaria coinfection and dengue with warning signs were more frequent among males. This pattern differs from reports from Medellín, Colombia, where a higher incidence of dengue has been described in males [16]. These differences may reflect geographic, contextual, or sociocultural variation and suggest the need to further explore biological and social factors that may modulate susceptibility and clinical response. In addition, sex-related differences raise questions about the possible role of sex hormones in the immune response to dengue infection [17]. A meta-analysis reported a slightly higher risk of severe dengue in females (OR: 1.13; 95% CI: 1.01–1.26), although this association was not observed in studies restricted to pediatric populations. Similarly, a study conducted in Mexico also described a higher risk of severe dengue among women [18,19].

Regarding age, children accounted for the highest proportion of severe dengue cases, whereas adults accounted for the highest proportion of dengue cases with warning signs. Coinfected cases were mainly distributed between childhood and adolescence. These findings are consistent with the epidemiological profile described in Colombia, where severe dengue occurs more frequently in children and adolescents, as well as in older adults, groups in whom plasma leakage and hemorrhagic complications require close clinical monitoring [20]. Although the low frequency in some age categories limited more robust comparisons, the age-group distribution observed in this series remains clinically informative.

The time from symptom onset to medical consultation was similar across the analyzed groups. This finding suggests that the initial clinical course may be comparable regardless of subsequent severity. Previous reports indicate that severe dengue usually becomes evident after the first days of illness, generally within a clinical window of 3 to 10 days [21]. This temporal framework is consistent with our observations, although no relevant variation in consultation time according to clinical classification was identified.

The geographic distribution of cases also revealed relevant regional patterns. San Jorge had the highest number of cases and the highest proportion of dengue–malaria coinfection, whereas Alto Sinú, Sinú Medio, and Urabá accounted for a greater proportion of severe dengue cases. Thus, the observed heterogeneity was defined not only by variation in total case burden across subregions, but also by differences in clinical presentation. These findings suggest that local epidemiological differences across subregions may influence clinical presentation and may pose challenges for regional health care planning. In contrast, subregions such as Costanera, Bajo Sinú, Sabana, and Valle de Aburrá contributed fewer cases, suggesting a more limited contribution to the overall clinical burden observed in this series. Colombia remains a hyperendemic setting for dengue, characterized by sustained viral circulation and high population seroprevalence [22]. In this context, the clinical findings observed in severe dengue cases are consistent with the World Health Organization 2009 classification [23,24].

The clinical manifestations identified in this series reinforce the relevance of early recognition of warning signs and severity-related findings. Fever was documented in all patients, while edema and elevated hematocrit were particularly frequent among severe dengue cases, reflecting plasma leakage and hemodynamic compromise. In addition, myalgia showed significant differences across clinical groups, being more frequent in dengue with warning signs, whereas edema was markedly concentrated in severe dengue. These observations are consistent with previous reports describing mucosal bleeding, fluid accumulation, shock, gastrointestinal bleeding, thrombocytopenia, and elevated hematocrit as important markers of severe dengue [20,25,26]. In this series, elevated hematocrit, edema, severe plasma leakage, and shock were particularly relevant among severe cases, supporting their value as clinical indicators of progression.

In endemic regions where dengue and malaria coexist, coinfection represents a significant clinical challenge. In this series, most fatal coinfected cases were associated with *Plasmodium falciparum*, which may contribute to greater clinical deterioration because of overlapping manifestations such as fever, thrombocytopenia, plasma leakage, and organ dysfunction [27,28]. These findings highlight the need to consider simultaneous diagnoses in patients presenting with acute febrile syndrome in endemic areas, especially when severe findings or atypical clinical evolution are present.

In referral settings where dengue cases are managed and complicated malaria cases are referred from endemic municipalities, clinicians should maintain a high index of suspicion for malaria in patients with suspected or confirmed dengue who originate from malaria-endemic areas, present with atypical clinical deterioration, or show severe hematological or systemic involvement not fully explained by dengue alone. Previous studies have shown that malaria, dengue, and dengue–malaria coinfection may be difficult to distinguish clinically during the early phase of illness, reinforcing the need for laboratory confirmation. A positive malaria test does not exclude concomitant dengue, and a positive dengue NS1 antigen or PCR does not exclude malaria. In endemic areas, because clinical manifestations overlap and coinfection are well described, confirmation of one infection should not preclude evaluation for the other when illness is severe, atypical, or incompletely explained by a single diagnosis [7,8,9,10,13]. In hospitalized febrile patients from the Brazilian Amazon, coinfection was associated with higher rates of severe disease, deep bleeding, hepatomegaly, and jaundice. Studies in Tanzania and Yemen also reported that clinical and sociodemographic characteristics alone could not reliably differentiate malaria, dengue, and coinfected cases. These findings support timely parasitological evaluation in patients with acute febrile illness from malaria-endemic territories, particularly when severe manifestations or unfavorable clinical evolution are present [10,13,29].

From a pathophysiological perspective, dengue–malaria coinfection may result in greater clinical severity through overlapping immune activation, hematological disturbances, and vascular injury. Available evidence suggests that coinfected patients can exhibit distinct inflammatory profiles, with increased concentrations of mediators such as IFN-γ, IL-6, TNF, and CCL4 in some settings. However, other studies have shown immune patterns more consistent with dengue monoinfection, indicating that these responses may vary by population, timing of infection, and pathogen burden [29,30,31]. Coinfection has also been associated with marked hematological abnormalities, particularly severe thrombocytopenia and anemia, as well as higher frequencies of severe disease, bleeding manifestations, hepatomegaly, and jaundice [1,4,11,29,32,33,34]. Recent evidence further suggests that coinfection may generate a synergistic immunopathogenic state in which amplified cytokine responses, complement overactivation, and platelet dysfunction contribute to greater vascular instability and plasma leakage [29,35,36]. Although direct evidence of endothelial dysfunction in human coinfected patients remains limited, experimental data support the biological plausibility of amplified vascular injury, showing that DENV infection can upregulate endothelial adhesion molecules such as ICAM-1 and potentially enhance cytoadherence of *Plasmodium*-infected erythrocytes [37]. Thus, the plasma leakage, thrombocytopenia, hemodynamic compromise, and fatal outcomes observed in our series may reflect the combined effects of cytokine dysregulation, endothelial injury, complement activation, and platelet dysfunction [29,36]. However, cytokines, endothelial activation markers, and vascular permeability mediators were not measured in our study; therefore, these mechanisms should be interpreted as plausible explanations supported by previous literature rather than as direct findings from our cohort.

An additional and clinically important challenge in severe dengue–malaria coinfection is fluid management, since both diseases may require opposing hemodynamic strategies [38,39]. In severe dengue, the critical phase is characterized by marked plasma leakage due to increased vascular permeability, which requires prompt but carefully titrated fluid resuscitation to restore intravascular volume and prevent shock [38,40,41]. In contrast, severe malaria is primarily driven by microcirculatory obstruction and tissue hypoxia rather than true hypovolemia, and therefore a more restrictive fluid strategy is recommended to reduce the risk of pulmonary edema and excess mortality [39,42]. In coinfected patients, this creates a therapeutic dilemma: insufficient fluid replacement may worsen shock and organ hypoperfusion, whereas excessive fluid administration may precipitate pulmonary edema and respiratory deterioration [39]. Therefore, dengue–malaria coinfection requires individualized assessment of volume status, close monitoring of perfusion and hematocrit, careful evaluation of pulmonary status, and frequent reassessment as the clinical picture evolves to balance the competing risks of vascular collapse and fluid overload.

Mortality findings also warrant attention. Six of the ten fatal cases occurred in patients with dengue–malaria coinfection, and most deaths originated in Córdoba. Common manifestations among fatal cases included fever, retro-orbital pain, fatigue, and severe plasma leakage. Additional findings, such as thrombocytopenia, mucosal bleeding, edema, hemodynamic compromise, gastrointestinal bleeding, and organ failure, were documented in several patients. Although the study design does not allow causal inference about mortality risk, these findings underscore the clinical complexity of severe dengue and dengue–malaria coinfection in a referral setting and support the need for timely diagnosis and close monitoring in patients with signs of deterioration.

The apparent concentration of fatal outcomes among coinfected patients should be interpreted cautiously. In our series, six of the ten deaths occurred in patients with dengue–malaria coinfection; however, the study design and sample size do not allow estimation of excess mortality risk attributable to coinfection. Previous evidence from the Peruvian Amazon found that *Plasmodium*/DENV coinfection was not associated with worse disease than monoinfection and resembled DENV monoinfection in symptom frequency and immune marker profile. Differences between studies may reflect variation in study design, population characteristics, infecting *Plasmodium* species, diagnostic methods, disease severity at presentation, and referral patterns [31].

This study should be interpreted as a descriptive, hospital-based case series rather than an inferential epidemiological study. Its purpose was to characterize clinically complex presentations of dengue with warning signs, severe dengue, and dengue–malaria coinfection treated at a regional referral hospital, not to estimate population-level associations or causal effects. Comparisons between clinical groups were exploratory, and *p*-values should be interpreted cautiously given the limited sample size and the low frequency of some outcomes. Therefore, the observed patterns should be considered hypothesis-generating and require confirmation in larger, prospective, multicenter studies with standardized diagnostic and laboratory protocols.

This study has limitations inherent to its retrospective design, small sample size, and reliance on routine clinical records and institutional surveillance data. These factors may have led to incomplete documentation, referral bias, and possible diagnostic misclassification. The use of a curated institutional surveillance database limited reconstruction of the full inclusion and exclusion flow before the database was delivered to the investigators. Dengue diagnosis was based on routine serological testing for dengue antibodies, while individual IgM/IgG patterns were not consistently available, and NS1 antigen testing or RT-PCR were not part of routine diagnostic procedures. Malaria diagnosis and species identification relied on routine thick-smear microscopy, which is operator-dependent and less sensitive than PCR for detecting low-density infections. Under field conditions, its detection threshold is approximately 50–100 parasites/µL; therefore, submicroscopic infections may have been missed, potentially misclassifying some dengue-only cases and underestimating the true frequency of dengue–malaria coinfection [43,44]. Moreover, species identification and parasitemia quantification were not consistently documented and may be less reliable at low parasite densities [45], limiting our ability to assess their association with clinical severity and mortality. In addition, some clinical and laboratory parameters that could have helped characterize coinfection more comprehensively, including splenomegaly, jaundice, blood chemistry parameters, hemoglobin, and inflammatory markers, were not consistently available for all patients. Despite these limitations, this case series provides local clinical evidence on severe dengue and dengue–malaria coinfection in a referral hospital in an endemic region and highlights the importance of early diagnostic suspicion, careful monitoring, and malaria testing in patients with suspected dengue who originate from malaria-endemic municipalities or show atypical deterioration.

## 5. Conclusions

This case series shows that severe dengue and dengue–malaria coinfection present as clinically complex conditions in a referral hospital serving populations from dengue- and malaria-endemic territories in northern Colombia. Severe dengue was characterized by findings consistent with plasma leakage and hemodynamic compromise, whereas dengue–malaria coinfection accounted for a substantial proportion of fatal cases and was predominantly associated with *Plasmodium falciparum*. These findings underscore the importance of ongoing clinical and epidemiological surveillance in endemic regions where both diseases coexist, as well as the need for early recognition of severity signs, timely malaria testing, and appropriate management in referral settings.

## Figures and Tables

**Table 1:** Sociodemographic and baseline characteristics of the study population according to clinical classification.

Variable		Dengue–Malaria Coinfection *n* = 14 (%)	Dengue with Warning Signs *n* = 14 (%)	Severe Dengue *n* = 25 (%)	Total *n* = 53 (%)
Sex	Male	12 (85.7)	13 (92.9)	6 (24)	31 (58.5)
	Female	2 (14.3)	1 (7.1)	19 (76)	22 (41.5)
Age, median (IQR), years		14 (6–19)	18 (12–21)	12 (8–22)	—
Age group	Childhood (0–11)	5 (35.7)	3 (21.4)	12 (48)	20 (37.7)
	Adolescence (12–18)	5 (35.7)	4 (28.6)	3 (12)	12 (22.6)
	Adulthood (19–59)	3 (21.4)	6 (42.9)	9 (36)	18 (34.0)
	Older adults (>60)	1 (7.1)	1 (7.1)	1 (4)	3 (5.7)
Time from symptom onset to consultation, median (IQR), days		3.5 (3–4)	4 (3–4.75)	4 (3–5)	—
Department and subregion of origin					
Córdoba	Alto Sinú (Tierralta, Valencia)	1 (7.1)	2 (14.3)	6 (24)	9 (17)
	Bajo Sinú (Lorica)	1 (7.1)	—	—	1 (1.9)
	Costanera (Moñitos, San Bernardo del Viento)	1 (7.1)	2 (14.3)	—	3 (5.7)
	Sabana (Sahagún)	1 (7.1)	—	—	1 (1.9)
	San Jorge (San José de Uré, Puerto Libertador, Pueblo Nuevo, Montelíbano, La Apartada)	7 (50)	1 (7.1)	5 (20)	13 (24.5)
	Sinú Medio (Montería, Ciénaga de Oro)	1 (7.1)	3 (21.4)	5 (20)	9 (17)
Antioquia	Bajo Cauca (El Bagre, Caucasia)	1 (7.1)	2 (14.3)	2 (8)	5 (9.4)
	Urabá (San Pedro de Urabá, San Juan de Urabá, Chigorodó, Apartadó, Arboletes)	—	4 (28.6)	6 (24)	10 (18.9)
	Valle de Aburrá (Medellín)	1 (7.1)	—	1 (4)	2 (3.8)

Notes: The symbol “—” indicates that no cases were observed in that category. Age groups were classified according to the life-course categories established by the Colombian Ministry of Health and Social Protection. Percentages for categorical variables were calculated by column using the total number of cases in each clinical group as the denominator. Percentages in the total column were calculated using the total study population as the denominator (*n* = 53). IQR: interquartile range.

**Table 2:** Clinical manifestations according to clinical classification.

Clinical Manifestations	Clinical Feature	Dengue with Warning Signs *n* (%)	Severe Dengue *n* (%)	Dengue–Malaria Coinfection *n* (%)	Total *n* (%)	*p*-Value
Systemic	Fever	14 (100)	25 (100)	14 (100)	53 (100)	__
	Loss of appetite	12 (85.7)	17 (68)	11 (78.6)	40 (75.5)	0.5143
	Fatigue	7 (50)	10 (40.0)	10 (71.4)	27 (50.9)	0.2023
	Hypertension	1 (7.1)	3 (12)	2 (14.3)	6 (11.3)	1.0000
Neurological	Dizziness	7 (50)	15 (60)	4 (28.6)	27 (50.9)	0.1983
	Somnolence and irritability	13 (92.9)	21 (84)	13 (92.9)	47 (88.7)	0.6376
	Headache	12 (85.7)	19 (76)	12 (85.7)	43 (81.1)	0.7275
	Retro-orbital pain	10 (71.4)	15 (60)	10 (71.4)	35 (66)	0.8040
Musculoskeletal	Myalgia	13 (92.9)	9 (36)	9 (64.3)	31 (58.5)	0.0013
	Arthralgia	6 (42.9)	15 (60)	11 (78.6)	32 (60.4)	0.1479
Dermatological	Rash	4 (28.6)	16 (64)	5 (35.7)	25 (47.2)	0.0747
	Exanthema	5 (35.7)	6 (24)	7 (50)	18 (34)	0.2503
Gastrointestinal	Nausea	6 (42.9)	11 (44)	10 (71.4)	27 (50.9)	0.2616
	Abdominal pain	13 (92.9)	18 (72)	11 (78.6)	42 (79.2)	0.3784
	Vomiting	10 (71.4)	18 (72)	11 (78.6)	39 (73.6)	0.9235
	Diarrhea	5 (35.7)	13 (52)	5 (35.7)	23 (43.4)	0.5871
	Hepatomegaly	8 (57.1)	18 (72)	8 (57.1)	34 (64.2)	0.5716
	Gastrointestinal bleeding	—	6 (24)	1 (7.1)	7 (13.2)	0.0778
Hemorrhagic/Hematologic	Mucosal bleeding	9 (64.3)	21 (84)	10 (71.4)	40 (75.5)	0.3532
	Elevated hematocrit	6 (42.9)	20 (80)	10 (71.4)	36 (67.9)	0.0778
	Thrombocytopenia (<50,000/µL)	14 (100)	25 (100)	13 (92.9)	52 (98.1)	0.5342
Capillary leakage and hemodynamic compromise	Edema	1 (7.1)	19 (76.0)	5 (35.7)	25 (47.2)	0.0003
	Severe plasma leakage	—	24 (96.0)	8 (57.1)	32 (60.4)	—
	Hemodynamic compromise	—	21 (84.0)	9 (64.3)	30 (56.6)	—
	Dengue shock	—	13 (52.0)	2 (14.3)	15 (28.3)	—
Severe organ involvement	Severe organ involvement	—	19 (76.0)	9 (64.3)	28 (52.8)	—

Note: The symbol “—” indicates that no cases were observed in that category. Percentages were calculated by column using the total number of cases in each clinical group as denominator. *p*-values were obtained using Fisher’s exact test. Variables directly related to the definition of severe dengue were described separately and were not included in inferential comparisons.

**Table 3:** Clinical and epidemiological characteristics of deceased patients treated at a referral hospital in Montería, Córdoba.

Case	Year	Sex	Age (years)	Origin	Common Clinical Manifestations	Case-Specific Clinical Findings	Case Classification	*Plasmodium* Species	Cause of Death
1	2018	F	4	Puerto Libertador, Córdoba	Fever, retro-orbital pain, fatigue, severe plasma leakage	Nausea, loss of appetite, myalgia, somnolence and irritability, hepatomegaly, mucosal bleeding, edema, thrombocytopenia (<50,000/µL)	Coinfection	*P. falciparum* (4500 parasites/µL)	Hypovolemic shock
2	2018	M	17	Montería, Córdoba	Fever, retro-orbital pain, fatigue, severe plasma leakage	Nausea, loss of appetite, severe headache, myalgia, arthralgia, rash, dizziness, exanthema, abdominal pain, vomiting, irritability, hepatomegaly, mucosal bleeding, thrombocytopenia (<50,000/µL)	Coinfection	*P. falciparum* (4800 parasites/µL)	Organ failure
3	2018	F	15	Medellín, Antioquia	Fever, retro-orbital pain, fatigue, severe plasma leakage	Nausea, severe headache, myalgia, arthralgia, rash, dizziness, exanthema, abdominal pain, diarrhea, vomiting, irritability, mucosal bleeding, thrombocytopenia (<50,000/µL), gastrointestinal bleeding	Coinfection	*P. vivax* (1800 parasites/µL)	Hemorrhagic complication
4	2018	F	14	Sahagún, Córdoba	Fever, retro-orbital pain, fatigue, severe plasma leakage	Nausea, loss of appetite, severe headache, myalgia, arthralgia, rash, dizziness, exanthema, abdominal pain, vomiting, irritability, hepatomegaly, mucosal bleeding, edema	Coinfection	*P. falciparum* (3500 parasites/µL)	Pulmonary embolism
5	2018	M	5	Montelíbano, Córdoba	Fever, retro-orbital pain, fatigue, severe plasma leakage	Nausea, loss of appetite, severe headache, myalgia, arthralgia, dizziness, exanthema, abdominal pain, vomiting, irritability, hepatomegaly, mucosal bleeding, hypotension, thrombocytopenia (<50,000/µL), hepatic failure	Coinfection	*P. falciparum* (5000 parasites/µL)	Septic shock
6	2023	M	82	Puerto Libertador, Córdoba	Fever, retro-orbital pain, fatigue, severe plasma leakage	Loss of appetite, severe headache, myalgia, dizziness, exanthema, abdominal pain, vomiting, irritability, hepatomegaly, mucosal bleeding, thrombocytopenia (<50,000/µL)	Coinfection	*P. falciparum* (3000 parasites/µL)	Organ failure
7	2018	M	19	Montería, Córdoba	Fever, retro-orbital pain, fatigue, severe plasma leakage	Nausea, loss of appetite, severe headache, myalgia, arthralgia, rash, dizziness, abdominal pain, vomiting, irritability, hepatomegaly, hypertension, mucosal bleeding, edema, hemodynamic compromise, thrombocytopenia (<50,000/µL)	Severe dengue	N/A	Respiratory failure
8	2018	M	11	Valencia, Córdoba	Fever, retro-orbital pain, fatigue, severe plasma leakage	Abdominal pain, vomiting, irritability, edema, hemodynamic compromise, thrombocytopenia (<50,000/µL)	Severe dengue	N/A	Septic shock
9	2018	F	51	Montería, Córdoba	Fever, retro-orbital pain, fatigue, severe plasma leakage	Nausea, loss of appetite, severe headache, myalgia, arthralgia, rash, dizziness, abdominal pain, vomiting, irritability, hepatomegaly, hypertension, mucosal bleeding, edema, hemodynamic compromise, thrombocytopenia (<50,000/µL)	Severe dengue	N/A	Organ failure
10	2022	F	38	El Bagre, Antioquia	Fever, retro-orbital pain, fatigue, severe plasma leakage	Nausea, loss of appetite, severe headache, dizziness, exanthema, abdominal pain, irritability, mucosal bleeding, thrombocytopenia (<50,000/µL)	Severe dengue	N/A	Hypovolemic shock

Note: Common clinical manifestations correspond to findings documented in all fatal cases. Case-specific clinical findings include additional manifestations recorded individually in each patient. N/A: not applicable because no dengue–malaria coinfection was recorded for these cases.

## Data Availability

The data presented in this study are not publicly available due to ethical and privacy restrictions related to the use of medical records containing sensitive clinical information.

## Abbreviations

The following abbreviations are used in this manuscript:

WHO	World Health Organization
IQR	Interquartile range
ESE	State Social Enterprise
DENV	Dengue Virus

## References

[1] World Health Organization Enfermedades Transmitidas por Vectores. https://www.who.int/es/news-room/fact-sheets/detail/vector-borne-diseases.

[2] Poveda G. (2020). Concomitant malaria, dengue and COVID-19: An extraordinary challenge for Colombia’s public health system. Curr. Opin. Environ. Sustain..

[3] Cárdenas M.I.E., Herrera V.M., Montoya M.C.M., Parra A.L., Moncayo Z.M.Z., García J.P.F., Barraquer I.R., Centeno L.Á.V. (2020). Heterogeneity of dengue transmission in an endemic area of Colombia. PLoS Negl. Trop. Dis..

[4] Vásquez-Trujillo A., Cardona-Arango D., Segura-Cardona A.M., Parra-Henao G.J. (2020). Burden of dengue in the State of Meta, Colombia (2010–2016). Cad. Saúde Pública.

[5] Espinal M.A., Andrus J.K., Jauregui B., Waterman S.H., Morens D.M., Santos J.I., Horstick O., Francis L.A., Olson D. (2019). Emerging and reemerging aedes-transmitted arbovirus infections in the region of the americas: Implications for health policy. Am. J. Public Health.

[6] Gebremariam T.T., Schallig H.D.F.H., Kurmane Z.M., Danquah J.B. (2023). Increasing prevalence of malaria and acute dengue virus coinfection in Africa: A meta-analysis and meta-regression of cross-sectional studies. Malar. J..

[7] Cerilo-Filho M., Arouca M.d.L., Medeiros E.D.S., De Jesus M.C., Sampaio M.P., Reis N.F., Silva J.R., Baptista A.R., Storti-Melo L.M., Machado R.L. (2024). Worldwide distribution, symptoms and diagnosis of the coinfections between malaria and arboviral diseases: A systematic review. Mem. Inst. Oswaldo Cruz.

[8] Belem L.R.W., Yao R.K., Amara M.F., Taita A.V.W., Kaboré B.P., Kouadio K.W.U., Ibemgbo S.A., Sangaré I., Gomgnimbou M.K. (2026). Molecular detection and epidemiological characterization of dengue and malaria coinfection in the Guiriko region, Burkina Faso. Acta Trop..

[9] Rana B., Trianty L., Santoso M.S., Puspitasari A.M., Nisa F.A., Amalia R., Carlier L., Tetteh K., Noviyanti R., Sasmono R.T. (2026). Co-infections of malaria and dengue in Timika, a highly endemic malaria area in Central Papua, Indonesia. PLoS ONE.

[10] Abdul-Ghani R., Mahdy M.A.K., Alkubati S., Al-Mikhlafy A.A., Alhariri A., Das M., Dave K., Gil-Cuesta J. (2021). Malaria and dengue in Hodeidah city, Yemen: High proportion of febrile outpatients with dengue or malaria, but low proportion co-infected. PLoS ONE.

[11] Bhatt S., Gething P.W., Brady O.J., Messina J.P., Farlow A.W., Moyes C.L., Drake J.M., Brownstein J.S., Hoen A.G., Sankoh O. (2013). The global distribution and burden of dengue. Nature.

[12] Alonso P., Noor A.M. (2017). The global fight against malaria is at crossroads. Lancet.

[13] Kayange N.M., Malande O.O., Koliopoulos P., Gehring S., Groendahl B., Wajanga B., Msaki B., Revocatus B., Mshana S.E. (2024). Malaria and dengue fever in febrile children entering healthcare facilities in Mwanza, Tanzania. PLoS ONE.

[14] Helías Rodríguez R. Distribución Espacial de la Malaria en el Departamento de Córdoba en el Periodo 2015–2018. Bachelor’s Thesis.

[15] World Health Organization (2009). Dengue Guidelines for Diagnosis, Treatment, Prevention and Control: New Edition.

[16] Warnes C.M., Santacruz-Sanmartin E., Carrillo F.B., Velez I.D. (2021). Surveillance and Epidemiology of Dengue in Medellín, Colombia from 2009 to 2017. Am. J. Trop. Med. Hyg..

[17] Fuentes-Vallejo M. (2017). Space and space-time distributions of dengue in a hyper-endemic urban space: The case of Girardot, Colombia. BMC Infect. Dis..

[18] Sangkaew S., Ming D., Boonyasiri A., Honeyford K., Kalayanarooj S., Yacoub S., Dorigatti I., Holmes A. (2021). Risk predictors of progression to severe disease during the febrile phase of dengue: A systematic review and meta-analysis. Lancet Infect. Dis..

[19] Vicente C.R., Cerutti Junior C., Fröschl G., Romano C.M., Cabidelle A.S.A., Herbinger K.H. (2017). Influence of demographics on clinical outcome of dengue: A cross-sectional study of 6703 confirmed cases in Vitória, Espírito Santo State, Brazil. Epidemiol. Infect..

[20] Rojas E.M., Herrera V.M., Miranda M.C., Rojas D.P., Gomez A.M., Pallares C., Cobos S.M., Pardo L., Gelvez M., Paez A. (2019). Clinical Indicators of Fatal Dengue in Two Endemic Areas of Colombia: A Hospital-Based Case–Control Study. Am. J. Trop. Med. Hyg..

[21] Che Isa Z., Lim J.A., Ain A.M., Othman F.A., Kueh Y.C., Tew M.M., Masnan M.J., Ibrahim A. (2023). Clinical profiles and predictors of survival in severe dengue cases. Singap. Med. J..

[22] Ciuoderis K.A., Usuga J., Moreno I., Perez-Restrepo L.S., Flórez D.Y., Cardona A., Cloherty G.A., Berg M.G., Hernandez-Ortiz J.P., Osorio J.E. (2023). Characterization of Dengue Virus Serotype 2 Cosmopolitan Genotype Circulating in Colombia. Am. J. Trop. Med. Hyg..

[23] Peña-García V.H., Triana-Chávez O., Mejía-Jaramillo A.M., Díaz F.J., Gómez-Palacio A., Arboleda-Sánchez S. (2016). Infection Rates by Dengue Virus in Mosquitoes and the Influence of Temperature May Be Related to Different Endemicity Patterns in Three Colombian Cities. Int. J. Environ. Res. Public Health.

[24] Htun T.P., Xiong Z., Pang J. (2021). Clinical signs and symptoms associated with WHO severe dengue classification: A systematic review and meta-analysis. Emerg. Microbes Infect..

[25] Zhang H., Zhou Y.P., Peng H.J., Zhang X.H., Zhou F.Y., Liu Z.H., Chen X.G. (2014). Predictive Symptoms and Signs of Severe Dengue Disease for Patients with Dengue Fever: A Meta-Analysis. BioMed Res. Int..

[26] Haq F.U., Imran M., Aslam Z., Mukhtar F., Jabeen K., Chaudhry M., Rahman S.U., Muhammad N. (2023). Severity of Dengue Viral Infection Based on Clinical and Hematological Parameters among Pakistani Patients. Am. J. Trop. Med. Hyg..

[27] Tsheten T., Clements A.C.A., Gray D.J., Adhikary R.K., Wangdi K. (2021). Clinical features and outcomes of COVID-19 and dengue co-infection: A systematic review. BMC Infect. Dis..

[28] Montiel J., Zuluaga L.M., Aguirre D.C., Segura C., Tobon-Castanõ A., Vásquez A.M. (2020). Microscopic and submicroscopic Plasmodium infections in indigenous and non-indigenous communities in Colombia. Malar. J..

[29] Mendonça V.R.R., Andrade B.B., Souza L.C.L., Magalhães B.M.L., Mourão M.P.G., Lacerda M.V.G., Barral-Netto M. (2015). Unravelling the patterns of host immune responses in Plasmodium vivax malaria and dengue co-infection. Malar. J..

[30] Halsey E.S., Baldeviano G.C., Edgel K.A., Vilcarromero S., Sihuincha M., Lescano A.G. (2016). Symptoms and Immune Markers in Plasmodium/Dengue Virus Co-infection Compared with Mono-infection with Either in Peru. PLoS Negl. Trop. Dis..

[31] Kotepui M., Mala W., Kwankaew P., Kotepui K.U., Masangkay F.R., Wilairatana P. (2023). Distinct cytokine profiles in malaria coinfections: A systematic review. PLoS Negl. Trop. Dis..

[32] Epelboin L., Hanf M., Dussart P., Ouar-Epelboin S., Djossou F., Nacher M., Carme B. (2012). Is dengue and malaria co-infection more severe than single infections? A retrospective matched-pair study in French Guiana. Malar. J..

[33] Magalhães B.M.L., Siqueira A.M., Alexandre M.A.A., Souza M.S., Gimaque J.B., Bastos M.S., Figueiredo R.M.P., Melo G.C., Lacerda M.V.G., Mourão M.P.G. (2014). *P. vivax* Malaria and Dengue Fever Co-infection: A Cross-Sectional Study in the Brazilian Amazon. PLoS Negl. Trop. Dis..

[34] Kotepui M., Kotepui K.U. (2019). Prevalence and laboratory analysis of malaria and dengue co-infection: A systematic review and meta-analysis. BMC Public Health.

[35] Carr J.M., Cabezas-Falcon S., Dubowsky J.G., Hulme-Jones J., Gordon D.L. (2020). Dengue virus and the complement alternative pathway. FEBS Lett..

[36] Nascimento E.J.M., Hottz E.D., Garcia-Bates T.M., Bozza F., Marques E.T.A., Barratt-Boyes S.M. (2014). Emerging Concepts in Dengue Pathogenesis: Interplay between Plasmablasts, Platelets, and Complement in Triggering Vasculopathy. Crit. Rev. Immunol..

[37] da Cruz M.G.S., Dos Santos R.O., Sousa M.G.T., Costa F.T., de Lacerda M.V.G., Lopes S.C.P., Lalwani P. (2025). Impact of dengue virus infection on the cytoadherence of Plasmodium vivax-infected erythrocytes. Mem. Inst. Oswaldo Cruz.

[38] Simmons C.P., Farrar J.J., Chau N.v.V., Wills B. (2012). Dengue. N. Engl. J. Med..

[39] Kalkman L.C., Hänscheid T., Krishna S., Grobusch M.P. (2022). Fluid therapy for severe malaria. Lancet Infect. Dis..

[40] Wilder-Smith A., Schwartz E. (2005). Dengue in Travelers. N. Engl. J. Med..

[41] Wilder-Smith A., Ooi E.-E., Horstick O., Wills B. (2019). Dengue. Lancet.

[42] Ishioka H., Plewes K., Pattnaik R., Kingston H.W.F., Leopold S.J., Herdman M.T., Mahanta K., Mohanty A., Dey C., Alam S. (2020). Associations Between Restrictive Fluid Management and Renal Function and Tissue Perfusion in Adults With Severe Falciparum Malaria: A Prospective Observational Study. J. Infect. Dis..

[43] Rapp T., Amagai K., Sinai C., Basham C., Loya M., Ngasala S., Said H., Muller M.S., Chhetri S.B., Yang G. (2024). Microheterogeneity of Transmission Shapes Submicroscopic Malaria Carriage in Coastal Tanzania. J. Infect. Dis..

[44] Agarwal R., Choi L., Johnson S., Takwoingi Y. (2020). Rapid diagnostic tests for Plasmodium vivax malaria in endemic countries. Cochrane Database Syst. Rev..

[45] Daily J.P., Parikh S. (2025). Malaria. N. Engl. J. Med..

